# Analysis of Survival Among Adults With Early-Onset Colorectal Cancer in the National Cancer Database

**DOI:** 10.1001/jamanetworkopen.2021.12539

**Published:** 2021-06-16

**Authors:** En Cheng, Holly N. Blackburn, Kimmie Ng, Donna Spiegelman, Melinda L. Irwin, Xiaomei Ma, Cary P. Gross, Fred K. Tabung, Edward L. Giovannucci, Pamela L. Kunz, Xavier Llor, Kevin Billingsley, Jeffrey A. Meyerhardt, Nita Ahuja, Charles S. Fuchs

**Affiliations:** 1 Department of Chronic Disease Epidemiology, Yale School of Public Health, New Haven, Connecticut; 2 Department of Surgery, Yale School of Medicine, New Haven, Connecticut; 3 Department of Medical Oncology, Dana-Farber Cancer Institute, Boston, Massachusetts; 4 Yale Cancer Center, Smilow Cancer Hospital, New Haven, Connecticut; 5 Department of Biostatistics, Yale School of Public Health, New Haven, Connecticut; 6 Center for Methods in Implementation and Prevention Science, Yale School of Public Health, New Haven, Connecticut; 7 Section of General Internal Medicine, Department of Internal Medicine, Yale School of Medicine, New Haven, Connecticut; 8 Cancer Outcomes, Public Policy, and Effectiveness Research Center, Yale Cancer Center, New Haven, Connecticut; 9 Division of Medical Oncology, Department of Internal Medicine, The Ohio State University College of Medicine, Columbus; 10 The Ohio State University Comprehensive Cancer Center—Arthur G. James Cancer Hospital and Richard J. Solove Research Institute, Columbus; 11 Department of Nutrition, Harvard T.H. Chan School of Public Health, Boston, Massachusetts; 12 Department of Epidemiology, Harvard T.H. Chan School of Public Health, Boston, Massachusetts; 13 Division of Hematology, Department of Internal Medicine, Yale School of Medicine, New Haven, Connecticut; 14 Division of Medical Oncology, Department of Internal Medicine, Yale School of Medicine, New Haven, Connecticut; 15 Section of Digestive Diseases, Department of Internal Medicine, Yale School of Medicine, New Haven, Connecticut

## Abstract

**Importance:**

While increased adherence to colorectal cancer (CRC) screening guidelines in the US has been associated with significant reductions in cancer incidence in US individuals aged 50 years and older, the incidence of CRC among those aged younger than 50 years has been steadily increasing. Understanding the survival among individuals with early-onset CRC compared with those aged 50 years and older is fundamental to informing treatment approaches and understanding the unique biological distinctiveness within early-onset CRC.

**Objective:**

To characterize the overall survival for individuals with early-onset CRC.

**Design, Setting, and Participants:**

This cohort study used data from the National Cancer Database. Included individuals were ages 0 to 90 years and diagnosed with primary CRC from January 1, 2004, through December 31, 2015. Individuals diagnosed at ages 51 through 55 years were selected as the reference group and defined as later-onset CRC for this study. Individuals diagnosed at age 50 years were excluded to minimize an apparent screening detection bias at that age in our population, given that these individuals disproportionately presented with earlier stage. All statistical analyses were conducted from January 4, 2020, through December 26, 2020.

**Exposures:**

Early-onset CRC was defined as age younger than 50 years at diagnosis.

**Main Outcomes and Measures:**

Overall survival was assessed by Kaplan-Meier analysis and Cox proportional hazards regression.

**Results:**

Among 769 871 individuals with CRC (377 890 [49.1%] women; 636 791 White individuals [82.7%]), 353 989 individuals (46.0%) died (median [range] follow-up: 2.9 [0-14.0] years), 102 168 individuals (13.3%) had early-onset CRC, and 78 812 individuals (10.2%) had later-onset CRC. Individuals with early-onset CRC, compared with those diagnosed with CRC at ages 51 through 55 years, had a lower 10-year survival rate (53.6% [95% CI, 53.2%-54.0%] vs 54.3% [95% CI, 53.8%-54.8%]; *P* < .001) in unadjusted analysis. However, after adjustment for other factors associated with mortality, most notably stage, individuals with early-onset CRC had a lower risk of death compared with individuals diagnosed from ages 51 through 55 years (adjusted hazard ratio [HR], 0.95 [95% CI, 0.93-0.96]; *P* < .001). In the model adjusted for stage, the HR for individuals with early-onset CRC was 0.89 (95% CI, 0.88-0.90; *P* < .001). The survival advantage was greatest for individuals diagnosed at ages 35 through 39 years (adjusted HR, 0.88 [95% CI, 0.84-0.92]; *P* < .001) and stages I (adjusted HR, 0.87 [95% CI, 0.81-0.93]; *P* < .001) and II (adjusted HR, 0.86 [95% CI, 0.82-0.90]; *P* < .001) and was absent among those diagnosed at ages 25 years or younger and stages III through IV.

**Conclusions and Relevance:**

These findings suggest that there is a survival benefit for individuals with early-onset CRC compared with those diagnosed with CRC at later ages. Further study is needed to understand the underlying heterogeneity of survival among individuals with early-onset CRC by age and stage.

## Key Points

**Question:**

How do rates of survival associated with early-onset colorectal cancer (CRC) compare with survival rates associated with CRC diagnosed at older ages?

**Findings:**

In this cohort study among 769 871 individuals diagnosed with primary CRC, after primary adjustment for stage at diagnosis, individuals with early-onset CRC (ie, those diagnosed at age <50 years) had a survival advantage compared with individuals diagnosed from ages 51 through 55 years. In addition, the survival advantage appeared greatest for individuals diagnosed at ages 35 through 39 years and stages I through II.

**Meaning:**

These findings suggest that there is a survival benefit associated with early-onset CRC compared with later-onset CRC and reinforce the importance of early CRC detection in the younger population.

## Teaser

This cohort study compares the survival among individuals with early-onset vs later-onset colorectal cancer using data from the US National Cancer Database.

## Introduction

Among adults aged younger than 50 years in the US, colorectal cancer (CRC) represents the second most common cancer diagnosis and the third leading cause of cancer death.^1^ Although there is no unequivocal definition of early-onset CRC, age younger than 50 years appears to be a common criterion in the published literature.^2,3^ The use of this criterion is driven, in part, by the recommendations across US independent expert panels published in the past 2 decades for CRC screening at age 50 years for individuals with the mean risk level.^4,5,6,7,8,9,10,11^

Early-onset CRC (ie, CRC diagnosed at age <50 years) has been characterized by unique clinical, genetic, and epigenetic characteristics,^2,3^ and thus it may be associated with different survival from CRC diagnosed among individuals older than 50 years. Reported comparisons of the survival of individuals with early-onset CRC with survival of those diagnosed at older ages have been somewhat inconsistent.^12,13,14,15,16,17,18,19,20,21,22,23^ These findings may have been highly influenced by different definitions for the comparison group of older individuals with CRC (eg, ages ≥50, 50-75, 60-80, >65, or 65-75 years).^12,13,14,15,16,17,18,19,20,21,22,23^ Inclusion of individuals aged 60 years and older in the comparison can introduce substantial mortality events unrelated to CRC diagnosis, thereby complicating these analyses.^24^ Thus, an ideal comparison group may be individuals diagnosed at or shortly after age 50 years, such as those aged 50 to 55 years, especially when CRC-specific survival could not be calculated owing to unavailability of causes of death. However, a 2020 study^25^ in the US Surveillance, Epidemiology, and End Results registries reported a steep incidence increase from age 49 to 50 years, consistent with previously undetected CRCs diagnosed through recommended screening uptake at age 50 years. In addition to this steep incidence increase, we hypothesized that CRC diagnosed specifically at age 50 years may have a higher proportion of early stage at diagnosis than CRC diagnosed at most ages. Therefore, diagnosis at age 50 years may have superior survival in all ages (ie, ages 0-90 years) of the study population owing to more intensive screening. Accordingly, we excluded individuals diagnosed at age 50 years from the comparison group and selected individuals diagnosed with CRC at ages 51 through 55 years to compare the survival differences for individuals diagnosed with early-onset CRC.

Additionally, individuals with early-onset CRC have been found to be more likely to be diagnosed at advanced stage.^2,3^ Therefore, we also hypothesized that stage at diagnosis would be associated with the comparison of survivals and may also contribute to heterogeneity of survival. Moreover, given that “younger than age 50 years” is a wide age range, there may be heterogeneity in early-onset CRC survival by age. Thus, we conducted the analyses using the National Cancer Database (NCDB), a large nationally representative cancer database, to verify our hypotheses of (1) superior survival associated with CRC diagnosis at age 50 years, (2) a survival advantage associated with early-onset CRC after primarily adjusting for stage, and (3) heterogeneity within early-onset CRC.

## Methods

Because NCDB data were deidentified, the Yale Institutional Review Board approved this cohort study as exempt human research and determined that informed consent was not required. This study followed the Strengthening the Reporting of Observational Studies in Epidemiology (STROBE) reporting guideline.

### Data Source and Study Population

Jointly sponsored by the Commission on Cancer (CoC) of the American College of Surgeons and the American Cancer Society, the NCDB is a clinical oncology database that captures 70% or more of new cancer diagnoses in the US and undergoes strict monitoring to ensure data quality and completeness.^26,27^ The NCDB and participating hospitals have not verified and are not responsible for the statistical validity of the data analysis or the conclusions derived by the authors in this study.

The NCDB was analyzed for individuals diagnosed with primary CRC from January 1, 2004, through December 31, 2015, and a total of 1 191 917 individuals were eligible initially. We excluded 295 621 individuals with a concomitant diagnosis or history of other malignant tumors, 54 445 individuals with noninvasive adenocarcinoma histologic examination findings, 71 891 individuals whose cancer staging results were unknown or not applicable based on the American Joint Commission on Cancer *Cancer Staging Manual*,^28,29^ and 89 individuals who were missing survival time. The derivation of the final study population of 769 871 individuals is presented in eFigure 1 in the Supplement.

### Study Population Characteristics

Characteristics of individuals in the study population were collected. These included age at diagnosis, sex (ie, male or female), race (ie, White, Black, or other), geographic location (ie, Northeast, Midwest, South, West, or other locations), residence setting (ie, metro, rural, urban, or unknown), median income in zip code of residence by quartiles (ie, <$30 000, $30 000-$34 999, $35 000-$45 999, ≥$46 000, or unknown), percentage of residents by zip code graduating from high school (ie, ≤71.0%, 71.1%-80.0%, 80.1%-86.0%, ≥86.1%, or unknown), primary health insurance (ie, none or unknown, private, Medicaid, or Medicare or other government insurance), tumor stage (ie, I, II, III, or IV), tumor location (ie, colon or rectum), Charlson-Deyo Comorbidity Index (CCI) score (ie, 0, 1, 2, or ≥3), facility type (ie, community cancer program or other community program, comprehensive community cancer program, academic or research program, or integrated network cancer program), surgical treatment (ie, yes, no, or unknown), radiation (ie, yes, no, or unknown), chemotherapy (ie, yes, no, or unknown), and immunotherapy (ie, yes, no, or unknown). Race was identified by electronic medical records at each registry that was included in the database. Race was commonly collected by medical records at each registry.

### Early-Onset CRC, Later-Onset CRC, and Overall Survival

Early-onset CRC was defined as CRC diagnosed at age younger than 50 years. The comparison group consisted of individuals diagnosed with CRC at ages 51 through 55 years, and this age group could be defined as later-onset CRC for this analysis. Individuals diagnosed at age 50 years were excluded to minimize an apparent screening detection bias at age 50 years in our population, given that these individuals disproportionately presented with earlier stages. Overall survival was the primary outcome of interest and was defined as the time from cancer diagnosis until death or the date of last contact.

### Statistical Analysis

In addition to descriptive analysis of individuals by age group, the characteristics of individuals with early-onset and later-onset CRC were compared using χ^2^ tests for categorical variables and the Mann-Whitney U test for continuous variables.^30,31^ Differences in overall survival between early-onset and later-onset CRC were assessed via the Kaplan-Meier method and the log-rank test,^32,33^ and we further conducted these analyses by stage.

We applied Cox proportional hazards regression to calculate hazard ratios (HRs) of overall mortality associated with age at diagnosis.^34,35^ To confirm the superior survival of individuals diagnosed with CRC at age 50 years in all ages (ie, ages 0-90 years), we first analyzed age principally by 1-year increments. Some years were combined owing to sparse data or homogeneity in measures of associations. For sensitivity analysis, we further excluded individuals aged 50 years (as stated previously) and younger than 10 years (owing to sparse data) and conducted restricted cubic spline regression to flexibly model the association of age at diagnosis with overall mortality.^36,37^

Regarding previous literature and biologic plausibility, the following covariates were evaluated as potential predictors associated with mortality: (1) demographic characteristics, such as sex, race, geographic location, and residence setting; (2) socioeconomic status, including median household income in zip code of residence by quartiles, percentage of residents by zip code graduating from high school, and primary health insurance; (3) clinical factors, such as tumor stage, tumor location, and CCI score; and (4) treatment factors, including facility type and treatment with surgical procedure, radiation, chemotherapy, and immunotherapy. Missing data for categorical variables were set as a level of unknown, whereas there were no missing data for continuous variables. Different sets of potential confounders, including or excluding stage, were adjusted for modeling.

Additionally, to explore heterogeneity in survival among individuals with early-onset CRC by age and stage, we further segmented the age at diagnosis of individuals with early-onset CRC into 5-year intervals and assessed the interactions of age at diagnosis (ie, <50 vs 51-55 years) with stage using stratified analyses with maximum partial likelihood tests.^38^ The Schoenfeld residuals method was used to test the proportional hazards assumption, and no violation was detected.^39^

All statistical analyses were conducted using SAS statistical software version 9.4 (SAS Institute) and R statistical software version 4.0.2 (R Project for Statistical Computing) from January 4, 2020, through December 26, 2020. All *P* values were 2-sided, and point estimates were presented with 95% CIs. The significance level was set at *P* = .05 for all analyses. We did not adjust for multiple comparisons.

## Results

Among 769 871 individuals with CRC (377 890 [49.1%] women; 636 791 White individuals [82.7%]), 353 989 individuals [46.0%] died (median [range] follow-up, 2.9 [0-14.0] years), 102 168 individuals (13.3%) had early-onset CRC, and 78 812 individuals (10.2%) had later-onset CRC. Individuals with early-onset CRC, compared with individuals diagnosed with CRC at ages 51 through 55 years, were more likely to be female (48 345 [47.3%] women vs 34 546 [43.8%] women; *P* < .001), be members of races in the other category (7004 individuals [6.9%] vs 4649 individuals [5.9%]; *P* < .001), have Medicaid (12 557 individuals [12.3%] vs 8087 individuals [10.3%]; *P* < .001), be diagnosed at an advanced stage (eg, 28 378 individuals [27.8%] vs 18 967 individuals [24.1%] with stage IV cancer; *P* < .001), have rectal tumors (29 983 individuals [29.3%] vs 22 643 individuals [28.7%]; *P* = .004), and use cancer treatment (ie, radiation: 25 277 [24.7%] vs 17 382 individuals [22.1%]; *P* < .001; chemotherapy: 69 451 individuals [68.0%] vs 46 673 individuals [59.2%]; *P* < .001; immunotherapy: 3356 individuals [3.3%] vs 2151 individuals [2.7%]; *P* < .001; and surgical treatment: 88 266 individuals [86.4%] vs 68 439 individuals [86.8%]; *P* = .005) and were less likely to have comorbidities (eg, 90 389 individuals [88.5%] vs 64 024 individuals [81.2%] with CCI score = 0; *P* < .001). Additional descriptive information for individuals by age group can be found in Table 1.

**Table 1.** Demographic and Clinical Characteristics of Individuals by Age at Diagnosis

Characteristic^a^	Age group, No. (%)				
	50 y (n = 16 365)	Early-onset CRC vs later-onset CRC			>55 y (n = 572 526)
		<50 y (n = 102 168)	51-55 y (n = 78 812)	*P* value^b^	
Died	4488 (27.4)	34 527 (33.8)	25 841 (32.8)	<.001	289 133 (50.5)
Follow-up time, median (IQR), y	3.6 (1.6-6.5)	3.3 (1.5-6.2)	3.4 (1.5, 6.3)	<.001	2.7 (0.9-5.7)
Sex					
Women	7449 (45.5)	48 345 (47.3)	34 546 (43.8)	<.001	287 550 (50.2)
Men	8916 (54.5)	53 823 (52.7)	44 266 (56.2)		284 976 (49.8)
Race					
White	12 827 (78.4)	79 813 (78.1)	61794 (78.4)	<.001	482 357 (84.3)
Black	2530 (15.5)	15 351 (15.0)	12369 (15.7)		62 843 (11.0)
Other	1008 (6.2)	7004 (6.9)	4649 (5.9)		27 326 (4.8)
Geographic location					
Northeast	3411 (20.8)	15 009 (14.7)	15 490 (19.7)	<.001	121 949 (21.3)
Midwest	4124 (25.2)	18 051 (17.7)	19 889 (25.2)		151 683 (26.5)
South	6228 (38.1)	30 280 (29.6)	31 312 (39.7)		215 119 (37.6)
West and other locations	2602 (15.9)	38 828 (38.0)	12 121 (15.4)		83 775 (14.6)
Residence setting					
Metro	13 461 (82.3)	84 098 (82.3)	64 094 (81.3)	<.001	459 419 (80.2)
Urban	2234 (13.7)	1724 (1.7)	1441 (1.8)		86 171 (15.1)
Rural	260 (1.6)	13 587 (13.3)	11 319 (14.4)		12 713 (2.2)
Unknown	410 (2.5)	2759 (2.7)	1958 (2.5)		14 223 (2.5)
Median income, %					
<30 000	2116 (12.9)	13 627 (13.3)	11120 (14.1)	<.001	79 161 (13.8)
30 000-34 999	2563 (15.7)	16 802 (16.5)	13432 (17.0)		103 093 (18.0)
35 000-45 999	4247 (26.0)	26 392 (25.8)	20786 (26.4)		155 874 (27.2)
≥46 000	6964 (42.6)	42 094 (41.2)	31079 (39.4)		218 220 (38.1)
Unknown	475 (2.9)	3253 (3.2)	2395 (3.0)		16 178 (2.8)
High school graduation rate, %					
≤71.0	2856 (17.5)	18 656 (18.3)	14 592 (18.5)	<.001	98 661 (17.2)
71.1-80.0	3473 (21.2)	23 035 (22.6)	18 053 (22.9)		134 046 (23.4)
80.1-86.0	3662 (22.4)	22 058 (21.6)	17 435 (22.1)		133 657 (23.4)
≥86.1	5897 (36.0)	35 149 (34.4)	26 331 (33.4)		189 928 (33.2)
Unknown	477 (2.91)	3270 (3.2)	2401 (3.1)		2774 (0.5)
Primary health insurance					
None or unknown	1398 (8.54)	10 953 (10.7)	7943 (10.1)	<.001	24 540 (4.3)
Private	12 304 (75.18)	73 125 (71.6)	56 283 (71.4)		157 200 (27.5)
Medicaid	1522 (9.3)	12 557 (12.3)	8087 (10.3)		21 551 (3.8)
Medicare or other government insurance	1141 (6.97)	5533 (5.4)	6499 (8.3)		369 235 (64.5)
Stage					
I	4876 (29.8)	19 905 (19.5)	20 287 (25.7)	<.001	137 765 (24.1)
II	3277 (20.02)	21 142 (20.7)	16 873 (21.4)		156 873 (27.4)
III	4743 (28.98)	32 743 (32.0)	22 685 (28.8)		156 133 (27.3)
IV	3469 (21.2)	28 378 (27.8)	18 967 (24.1)		121 755 (21.3)
Tumor location					
Colon	11 374 (69.5)	72 185 (70.7)	56 169 (71.3)	.004	459 720 (80.3)
Rectum	4991 (30.5)	29 983 (29.3)	22 643 (28.7)		112 806 (19.7)
CCI score					
0	13 748 (84.0)	90 389 (88.5)	64 024 (81.2)	<.001	386 820 (67.6)
1	2101 (12.8)	9795 (9.6)	11 767 (14.9)		130 339 (22.8)
2	351 (2.1)	1293 (1.3)	2052 (2.6)		38 437 (6.7)
≥3	165 (1.0)	691 (0.7)	969 (1.2)		16 930 (3.0)
Facility type					
Community cancer program or other community program	1656 (10.1)	34 571 (33.8)	8278 (10.5)	<.001	70260 (12.3)
Comprehensive community cancer program	6685 (40.9)	29 967 (29.3)	32 631 (41.4)		262 487 (45.9)
Academic or research program	5729 (35.0)	27 132 (26.6)	26 672 (33.8)		157 105 (27.4)
Integrated network cancer program	2295 (14.0)	10 498 (10.3)	11 231 (14.3)		82 674 (14.4)
Surgical treatment					
Yes	14 542 (88.9)	88 266 (86.4)	68 439 (86.8)	.005	493 040 (86.1)
No	1808 (11.1)	13 777 (13.5)	10 301 (13.1)		78 939 (13.8)
Unknown	15 (0.1)	125 (0.1)	72 (0.1)		547 (0.1)
Radiation					
Yes	3548 (21.7)	25 277 (24.7)	17382 (22.1)	<.001	82 006 (14.3)
No	12 680 (77.5)	75 980 (74.4)	60688 (77.0)		485 249 (84.8)
Unknown	137 (0.9)	911 (0.9)	742 (0.9)		5271 (0.9)
Chemotherapy					
Yes	9549 (58.4)	69 451 (68.0)	46 673 (59.2)	<.001	231 369 (40.4)
No	6348 (38.8)	29 607 (29.0)	29 491 (37.4)		318 744 (55.7)
Unknown	468 (2.9)	3110 (3.0)	2648 (3.4)		22 413 (3.9)
Immunotherapy					
Yes	397 (2.4)	3356 (3.3)	2151 (2.7)	<.001	8493 (1.5)
No	15 781 (96.4)	97 678 (95.6)	75 754 (96.1)		557 590 (97.4)
Unknown	187 (1.1)	1134 (1.1)	907 (1.2)		6443 (1.13)

Abbreviations: CCI, Charlson-Deyo Comorbidity Index; CRC, colorectal cancer; IQR, interquartile range.

^a^ Continuous variables were presented as median (IQR) and categorical variables as No. (%). Percentages may not add up to 100% owing to rounding.

^b^ *P* values were calculated using the χ^2^ test for categorical variables and Mann-Whitney U test for continuous variables.

**Figure 1.**
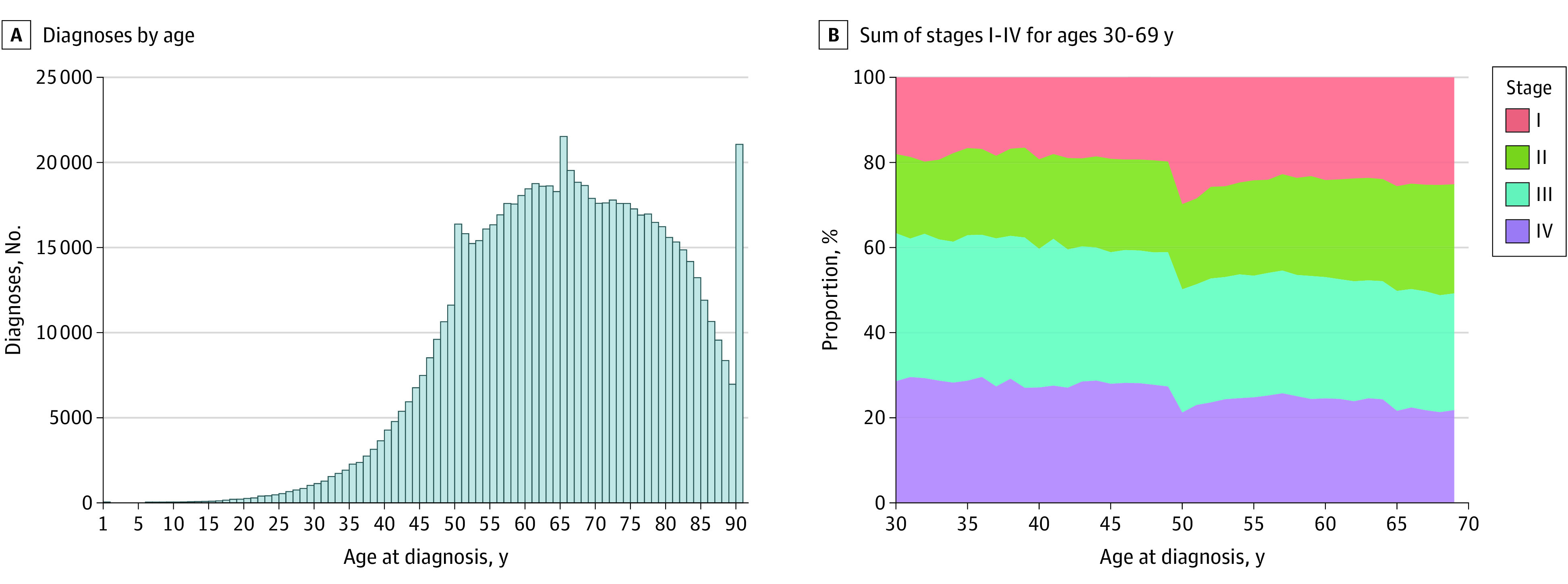
Distribution of Age at Diagnosis and Stage Among Individuals With Colorectal Cancer in the National Cancer Database Part B, the sum of stages I through IV for each age was 100%.

In defining the appropriate reference group, we considered the increasing body of evidence for an apparent screening detection bias at age 50 years, reflecting colorectal cancer screening guidelines during the time frame of our analysis.^4,5,6,7,8,9,10,11,25^ We assessed incident CRC diagnoses by 1-year age increments across the study cohort. Compared with the 1-year prior age, an increase was observed at the transition at age 50 years (Figure 1A), from 11 597 CRC diagnoses at age 49 years to 16 365 diagnoses at age 50 years, which represented 41.1% increase in incidence. In contrast, although the number of incident CRC diagnoses increased continuously until age 65 years, the mean 1-year increase in incident CRC diagnoses was 11.5% (95% CI, 10.9%-12.4%) for ages 45 to 49 years. We further assessed stage at diagnosis across 1-year age increments, finding a transient step-up in earlier stage (ie, I and II) at age 50 years. Specifically, 2296 of 11 597 individuals with CRC at age 49 years (19.8%) and 4876 of 16 365 individuals with CRC at age 50 years (29.8%) had stage I disease, reflecting a 50.5% relative increase at this transition (Figure 1B; eTable 1 in the Supplement). Additionally, we assessed overall survival by 1-year increments in all ages, observing a decrease in mortality risk for individuals diagnosed at age 50 years (eTable 2 in the Supplement), which was also verified by the restricted cubic splines plot (eFigure 2 in the Supplement). Therefore, based on these findings, we excluded individuals diagnosed at age 50 years from the referent group and selected individuals with CRC diagnosed at ages 51 to 55 years as the comparison group.

### Primary Outcome

**Figure 2.**
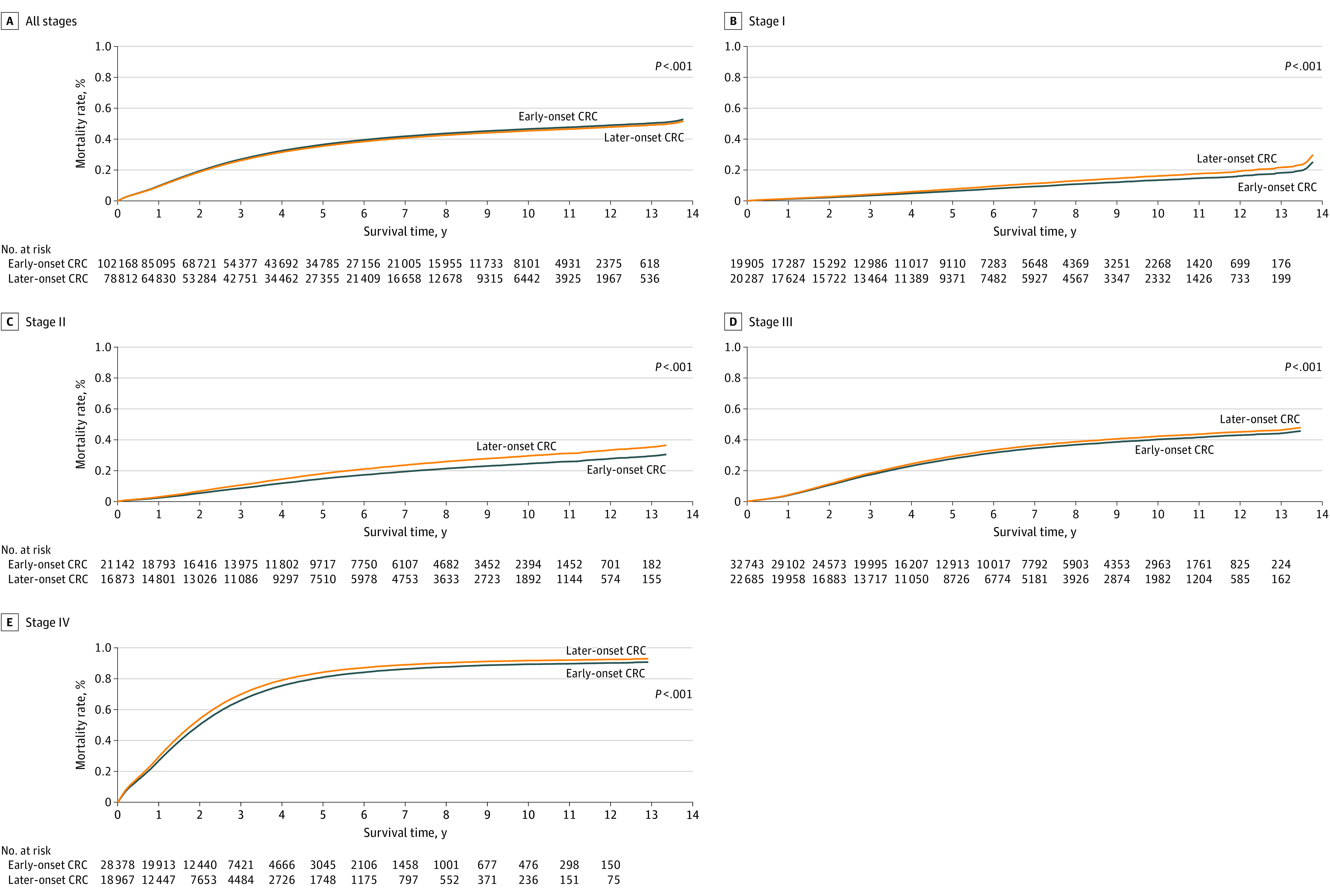
Kaplan-Meier Mortality Estimates for Early-Onset and Later-Onset Colorectal Cancer (CRC) by Stage Overall mortality of early-onset and later-onset CRC by stage is listed for 180 980 individuals at all stages, 40 192 individuals at stage I, 38 015 individuals at stage II, 55 428 individuals at stage III, and 47 345 individuals at stage IV. The *P*-value was calculated using the log-rank test.

Compared with individuals diagnosed with CRC at ages 51 to 55 years, individuals with early-onset CRC experienced inferior overall survival (log-rank *P* < .001) for all years (Figure 2A). Specifically, compared with individuals diagnosed with CRC from ages 51 to 55 years, individuals with early-onset CRC had a lower 10-year survival rate (53.6% [95% CI, 53.2%-54.0%] vs 54.3% [95% CI, 53.8%-54.8%]; *P* < .001) in the unadjusted Kaplan-Meier analysis (eTable 3 in the Supplement). However, stratified by stage, individuals with early-onset CRC had higher survival rates across all years of follow-up (Figure 2B-E; eTable 3 in the Supplement). The corresponding adjusted HRs for stages I to IV were 0.87 (95% CI, 0.81-0.93; *P* < .001), 0.86 (95% CI, 0.82-0.90; *P* < .001), 0.98 (95% CI, 0.95-1.01; *P* = .15), and 0.96 (95% CI, 0.94-0.98; *P* < .001).

We further examined survival differences between early-onset and later-onset CRC via adjusting for other predictors associated with mortality (Table 2). Compared with individuals diagnosed at ages 51 to 55 years, the unadjusted HR for overall mortality among individuals with early-onset CRC was 1.04 (95% CI, 1.02-1.05; *P* < .001). However, with progressive multivariable adjustment, most notably with adjustment for stage, individuals with early-onset CRC had a reduction in mortality. Within the fully adjusted model, the multivariable HR for overall mortality for individuals with early-onset CRC was 0.95 (95% CI, 0.93-0.96; *P* < .001) compared with individuals diagnosed from age 51 to 55 years. In the model adjusted for stage, the HR for individuals with early-onset CRC was 0.89 (95% CI, 0.88-0.90; *P* < .001).

**Table 2.** Overall Mortality Comparing Early-Onset and Later-Onset CRC^a^

Age, y	Unadjusted		Model 1^b^		Model 2^c^		Model 3^d^	
	HR (95% CI)	*P* value	HR (95% CI)	*P* value	HR (95% CI)	*P* value	HR (95% CI)	*P* value
Early-onset vs later-onset CRC								
<50	1.04 (1.02-1.05)	<.001	0.89 (0.88-0.90)	<.001	1.02 (1.00-1.03)	.08	0.95 (0.93-0.96)	<.001
51-55	1 [Reference]	NA	1 [Reference]	NA	1 [Reference]	NA	1 [Reference]	NA
Early-onset vs later-onset CRC by age group								
<20	0.83 (0.71-0.97)	.02	1.08 (0.93-1.26)	.32	1.05 (0.90-1.23)	.53	1.04 (0.88-1.22)	.66
20-24	1.04 (0.95-1.14)	.36	1.08 (0.99-1.18)	.09	1.05 (0.96-1.16)	.29	1.07 (0.97-1.17)	.17
25-29	1.07 (1.01-1.14)	.02	0.93 (0.88-0.99)	.02	1.01 (0.94-1.08)	.76	0.94 (0.88-1.00)	.07
30-34	1.05 (1.00-1.09)	.04	0.85 (0.82-0.89)	<.001	1.00 (0.95-1.06)	.93	0.90 (0.85-0.95)	<.001
35-39	0.99 (0.96-1.03)	.74	0.83 (0.80-0.85)	<.001	0.97 (0.93-1.02)	.22	0.88 (0.84-0.92)	<.001
40-44	1.02 (1.00-1.05)	.07	0.88 (0.86-0.90)	<.001	1.00 (0.98-1.03)	.69	0.94 (0.92-0.96)	<.001
45-49	1.05 (1.03-1.07)	<.001	0.91 (0.90-0.93)	<.001	1.02 (1.00-1.04)	.02	0.96 (0.94-0.98)	<.001
51-55	1 [Reference]	NA	1 [Reference]	NA	1 [Reference]	NA	1 [Reference]	NA

Abbreviations: CRC, colorectal cancer; HR, hazard ratio; NA, not applicable.

^a^ Demographic characteristics included sex, race, geographic location, and residence setting. Socioeconomical status included median income in zip code of residence by quartiles, percentage of residents by zip code graduating from high school, and primary health insurance. Clinical factors included stage, tumor location, and Charlson-Deyo Comorbidity Index score. Treatment factors included facility type and use of surgical treatment, radiation, chemotherapy, and immunotherapy.

^b^ Adjusted for stage.

^c^ Adjusted for demographic characteristics, socioeconomical status, clinical factors (not including stage), and treatment factors.

^d^ Adjusted for demographic characteristics, socioeconomical status, clinical factors (including stage), and treatment factors.

### Subgroup Analyses

We further assessed for heterogeneity in survival among individuals with early-onset CRC (Table 2). Compared with individuals diagnosed from age 51 to 55 years, individuals diagnosed from age 35 to 39 years had the greatest mortality reduction (adjusted HR, 0.88 [95% CI, 0.84-0.92]; *P* < .001). In contrast, individuals diagnosed from age 20 to 24 years (adjusted HR, 1.07 [95% CI, 0.97-1.17]; *P* = .17) and at age younger than 20 years (adjusted HR, 1.04 [95% CI, 0.88-1.22]; *P* = .66) did not experience a survival advantage.

We also examined stage-specific survival for individuals with early-onset CRC (eTable 4 in the Supplement). In multivariable analyses, the superior survival was limited to individuals diagnosed at stage I (adjusted HR, 0.87 [95% CI, 0.81-0.93]; *P* < .001) and II (adjusted HR, 0.86 [95% CI, 0.82-0.90]; *P* < .001). The test for statistical interaction between age at diagnosis and stage was statistically significant (*P* for interaction < .001).

## Discussion

In this cohort study using a large US database, we observed an increase in incident CRC diagnoses at age 50 years, with an increased proportion of individuals with earlier stage disease and a transient decrease in mortality. These findings motivated us to select individuals diagnosed with CRC at ages 51 to 55 years as the comparison group for early-onset CRC, and we found that individuals with early-onset CRC were more likely to be diagnosed at advanced stage and experienced inferior unadjusted overall survival rates. However, following adjustment for other predictors associated with mortality, most notably stage, we found that individuals with early-onset CRC had a superior survival compared with those diagnosed at ages 51 to 55 years. That advantage was greatest for individuals diagnosed from ages 35 to 39 years, however, and was largely limited to individuals diagnosed at stage I and II.

The increase in diagnoses from ages 49 to 50 years was likely associated with historical CRC screening guidelines in the US^4,5,6,7,8,9,10,11^ rather than any biological factor.^2,3^ This is further supported by our finding of an increased proportion of earlier stage disease among individuals diagnosed at age 50 years and is associated with an improved survival for individuals aged 50 years. Few studies have specifically investigated the survival of individuals diagnosed with CRC at age 50 years, and our findings may suggest a higher burden of undetected preclinical early-onset CRC among individuals aged younger than 50 years, especially those ages 45 to 49 years. We would hypothesize that CRC diagnosed at ages 45 to 49 years may not be biologically different from CRC diagnosed at age 50 years.^2,3^ Thus, we would expect similar survival advantages from screening in the age range of 45 to 49 years. Recently, the draft CRC screening guideline of the US Preventive Services Task Force recommended starting CRC screening at age 45 years.^40^ Our findings may have policy implications and may inform the current debate on whether to decrease the age of initial CRC screening.

Prior studies^12,13,14,15,16,17,18,19,20,21,22,23^ assessing the survival differences of individuals with early-onset CRC used different ages (ie, ≥50, 50-75, 60-80, >65, or 65-75 years) as comparison groups, which may partly explain the inconsistent findings across prior studies. In contrast, we selected a relatively comparable age group (ages 51-55 years) that excluded individuals diagnosed at age 50 years for comparison and reported worse survival rates in all years for individuals with early-onset CRC. However, after adjustment for stage at diagnosis, individuals with early-onset CRC actually had better survival, with a relative 5% reduction in mortality (adjusted HR, 0.95 [95% CI, 0.93-0.96]). This may reinforce the importance of early CRC detection in the younger population, especially given that we are in the midst of a shift in the recommended age for CRC screening.^40^ However, considering that younger people are generally healthier, with more years remaining to live,^24^ the survival advantage should be interpreted cautiously, especially given that the advantage has a small magnitude and is heterogeneous by ages and stage.

Early-onset CRC is often characterized by more advanced stage, poorer cell differentiation, increased prevalence of mucosal and signet ring cell histology, left-sided (ie, distal colon and rectum) location of primary tumors, loss of DNA methylation, increased rate of *KRAS* and *TP53* mutations, and increased proportion of cancer family syndromes.^2,3,41,42^ These distinguishing characteristics suggest that early-onset CRC may exhibit unique biologic features and a potentially different prognosis when compared with CRC diagnosed among older individuals. Of note, we observed some heterogeneity in adjusted mortality risk for age subsets within the population with early-onset CRC. Specifically, the survival advantage was greatest among individuals diagnosed from ages 35 to 39 years and was absent among those diagnosed at age 25 years or younger. Interestingly, hereditary nonpolyposis colorectal cancer, owing to underlying mismatch repair deficiency, is associated with superior survival^43,44^ and is often diagnosed in individuals from ages 35 to 45 years.^45,46^ In contrast, adenomatous polyposis coli syndrome is more common in individuals who are diagnosed with CRC at age younger than 20 years (10%) compared with those diagnosed at later ages (0.1%),^47^ and adenomatous polyposis coli syndrome is not associated with a survival advantage.^48,49^ These high penetrance syndromes could partly account for the relative heterogeneity in survival across ages among individuals with early-onset CRC. Supportive of this hypothesis, previous findings also reported an increased prevalence of somatic mutations in *CTNNB1* among individuals with CRC who were younger than age 30 years and the highest proportion of consensus molecular subtype-1 (CMS-1; ie, microsatellite instability immune subtype) among individuals with CRC who were younger than age 40 years.^50,51^

### Limitations

This study has several limitations. First, since the NCDB did not collect information on screening, we could confirm that increases in diagnoses from age 49 to 50 years and the survival advantage associated with diagnosis at age 50 years necessarily reflected screening practices. Second, because data on causes of death were not available, we could not calculate CRC-specific mortality. Thus, to address the association of early-onset CRC with survival among young individuals, we selected a relatively young and comparably aged (ie, ages 51-55 years) CRC population for comparison. Third, given that there is limited information to distinguish curative vs palliative types of surgical treatment, chemotherapy, and radiotherapy in the NCDB, we could not fully address how treatment types (eg, curative vs palliative) may be associated with survival. Fourth, given that molecular signatures were unavailable in the NCDB, we could not verify biological distinctiveness within early-onset CRC in etiology and progression. However, our analysis of segmented early-onset CRC may encourage future molecular studies to investigate biological distinctiveness and heterogeneity in the early-onset CRC population. Fifth, given that this is an observation study, residual confounding cannot be ruled out.

## Conclusions

This study’s finding that younger individuals with CRC had increased mortality in unadjusted analyses, which was associated with being diagnosed at later stages of illness, suggests that more attention in screening given to younger individuals may reduce their mortality if their diseases can be detected at earlier stage. Our finding of a survival advantage associated with early-onset CRC among younger individuals should be interpreted cautiously, given that the advantage had a small magnitude and was heterogeneous by age and stage. Further study is needed to understand the underlying heterogeneity of survival by age and stage among individuals with early-onset CRC.

## Notes

Supplement. **eFigure 1.** Flow Diagram of Individuals With Colorectal Cancer in the National Cancer Database (January 1, 2004-December 31, 2015) Evaluated for Potential Enrollment **eTable 1.** Stage of Colorectal Cancer by Age at Diagnosis **eTable 2.** Hazard Ratios (95% CIs) of Age at Diagnosis With Overall Mortality **eFigure 2.** Associations of Age at Diagnosis With Overall Mortality **eTable 3.** Survival Rates (% With 95% CIs) of Early-Onset and Later-Onset Colorectal Cancer by Stage **eTable 4.** Multivariable Hazard Ratios Comparing Early-Onset vs. Later-Onset CRC Colorectal Cancer by Stage
