# Supplementary material for: Analysis of Survival Among Adults With Early-Onset Colorectal Cancer in the National Cancer Database
Source: JAMA Netw Open. 2021 Jun 16;4(6):e2112539. doi: 10.1001/jamanetworkopen.2021.12539 (PMC8209612; doi:10.1001/jamanetworkopen.2021.12539)
Supplement: Supplement. — eFigure 1. Flow Diagram of Individuals With Colorectal Cancer in the National Cancer Database (January 1, 2004-December 31, 2015) Evaluated for Potential Enrollment eTable 1. Stage of Colorectal Cancer by Age at Diagnosis eTable 2. Hazard Ratios (95% CIs) of Age at Diagnosis With Overall Mortality eFigure 2. Associations of Age at Diagnosis With Overall Mortality eTable 3. Survival Rates (% With 95% CIs) of Early-Onset and Later-Onset Colorectal Cancer by Stage eTable 4. Multivariable Hazard Ratios Comparing Early-Onset vs. Later-Onset CRC Colorectal Cancer by Stage [file jamanetwopen-e2112539-s001.pdf]

Supplemental Online Content

Cheng E, Blackburn HN, Ng K, et al. Analysis of survival among adults with early-onset colorectal cancer in the National Cancer Database. *JAMA Netw Open*. 2021;4(6):e2112539. doi:10.1001/jamanetworkopen.2021.12539

eFigure 1. Flow Diagram of Individuals With Colorectal Cancer in the National Cancer Database (January 1, 2004-December 31, 2015) Evaluated for Potential Enrollment

eTable 1. Stage of Colorectal Cancer by Age at Diagnosis

eTable 2. Hazard Ratios (95% CIs) of Age at Diagnosis With Overall Mortality

eFigure 2. Associations of Age at Diagnosis With Overall Mortality

eTable 3. Survival Rates (% With 95% CIs) of Early-Onset and Later-Onset Colorectal Cancer by Stage

eTable 4. Multivariable Hazard Ratios Comparing Early-Onset vs. Later-Onset CRC Colorectal Cancer by Stage

This supplemental material has been provided by the authors to give readers additional information about their work.

© 2021 Cheng E et al. *JAMA Network Open.*

eFigure 1. Flow Diagram of Individuals With Colorectal Cancer in the National Cancer Database (January 1, 2004-December 31, 2015) Evaluated for Potential Enrollment

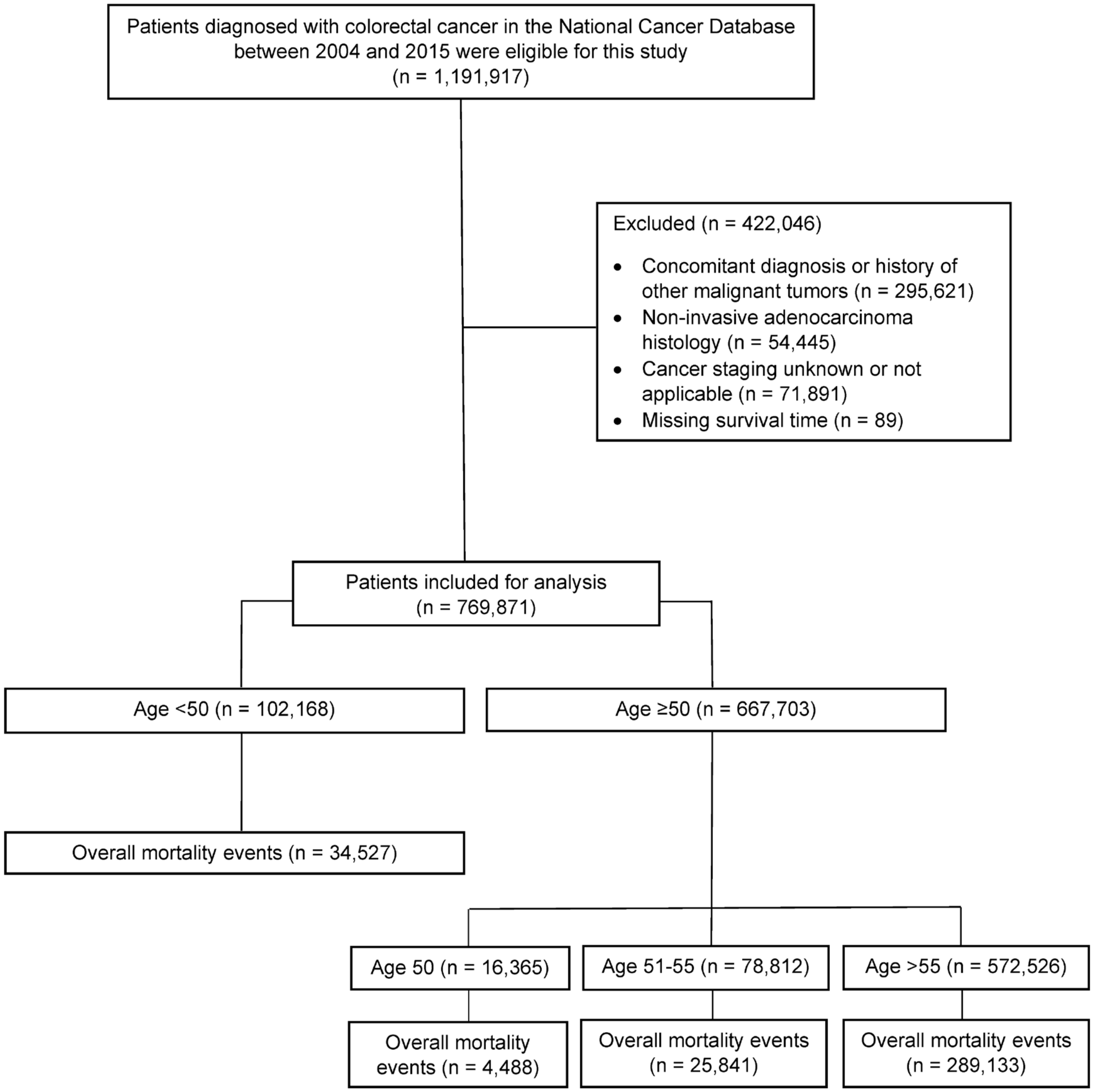

© 2021 Cheng E et al. *JAMA Network Open.*

eTable 1. Stage of Colorectal Cancer by Age at Diagnosis

		Stage[a]		
**Age**	I (%)	II (%)	III (%)	IV (%)
<20	392 (51.9)	78 (10.3)	169 (22.4)	116 (15.4)
20 - <25	542 (33.2)	253 (15.5)	461 (28.3)	376 (23.0)
25 - <30	839 (23.1)	642 (17.7)	1,167 (32.1)	983 (27.1)
30	197 (18.0)	203 (18.6)	380 (34.8)	312 (28.6)
31	231 (18.7)	237 (19.2)	402 (32.6)	365 (29.6)
32	299 (19.8)	256 (17.0)	512 (33.9)	442 (29.3)
33	328 (19.4)	317 (18.7)	562 (33.2)	486 (28.7)
34	336 (17.8)	392 (20.8)	624 (33.1)	532 (28.2)
35	372 (16.6)	458 (20.5)	765 (34.2)	642 (28.7)
36	394 (16.8)	472 (20.2)	782 (33.4)	692 (29.6)
37	500 (18.4)	526 (19.4)	944 (34.8)	741 (27.3)
38	522 (16.8)	637 (20.5)	1,045 (33.6)	907 (29.2)
39	597 (16.5)	763 (21.1)	1,276 (35.3)	976 (27.0)
40	817 (19.3)	892 (21.0)	1,382 (32.6)	1,149 (27.1)
41	856 (18.0)	945 (19.9)	1,637 (34.5)	1,306 (27.5)
42	1,015 (19.0)	1,149 (21.5)	1,738 (32.5)	1,447 (27.1)
43	1,126 (19.1)	1,221 (20.7)	1,877 (31.8)	1,684 (28.5)
44	1,251 (18.6)	1,443 (21.4)	2,106 (31.3)	1,934 (28.7)
45	1,429 (19.2)	1,637 (22.0)	2,306 (30.9)	2,086 (28.0)
46	1,643 (19.3)	1,807 (21.3)	2,655 (31.2)	2,394 (28.2)
47	1,852 (19.3)	2,048 (21.4)	2,988 (31.2)	2,693 (28.1)
48	2,071 (19.5)	2,296 (21.6)	3,305 (31.1)	2,944 (27.7)
49	2,296 (19.8)	2,470 (21.3)	3,660 (31.6)	3,171 (27.3)
50	4,876 (29.8)	3,277 (20.0)	4,743 (29.0)	3,469 (21.2)
51	4,501 (28.5)	3,184 (20.2)	4,485 (28.4)	3,633 (23.0)
52	3,917 (25.7)	3,278 (21.5)	4,434 (29.1)	3,589 (23.6)
53	3,942 (25.6)	3,284 (21.3)	4,421 (28.7)	3,747 (24.3)

© 2021 Cheng E et al. *JAMA Network Open.*

54	3,976 (24.7)	3,470 (21.6)	4,678 (29.1)	3,951 (24.6)
55	3,951 (24.2)	3,657 (22.4)	4,667 (28.6)	4,047 (24.8)
56	4,071 (24.1)	3,702 (21.9)	4,874 (28.8)	4,262 (25.2)
57	3,999 (22.8)	3,984 (22.7)	5,072 (28.9)	4,523 (25.7)
58	4,146 (23.6)	4,002 (22.8)	5,000 (28.5)	4,394 (25.1)
59	4,190 (23.2)	4,235 (23.5)	5,220 (28.9)	4,402 (24.4)
60	4,452 (24.2)	4,204 (22.8)	5,258 (28.5)	4,523 (24.5)
61	4,497 (24.0)	4,398 (23.5)	5,276 (28.1)	4,575 (24.4)
62	4,419 (23.8)	4,492 (24.2)	5,244 (28.2)	4,436 (23.9)
63	4,400 (23.6)	4,482 (24.1)	5,158 (27.7)	4,569 (24.6)
64	4,371 (23.9)	4,385 (24.0)	5,076 (27.8)	4,444 (24.3)
65	5,506 (25.6)	5,295 (24.6)	6,071 (28.2)	4,647 (21.6)
66	4,879 (25.0)	4,836 (24.8)	5,436 (27.8)	4,373 (22.4)
67	4,753 (25.3)	4,715 (25.1)	5,260 (27.9)	4,096 (21.8)
68	4,712 (25.3)	4,831 (25.9)	5,121 (27.5)	3,968 (21.3)
69	4,494 (25.1)	4,583 (25.6)	4,908 (27.5)	3,892 (21.8)
70 - <75	22,457 (25.5)	23,688 (26.9)	23,923 (27.2)	18,061 (20.5)
75 - <80	20,910 (25.0)	24,627 (29.4)	22,313 (26.6)	15,938 (19.0)
>80	31,509 (22.3)	46,414 (32.8)	36,923 (26.1)	26,652 (18.8)

[a] Cancer stage was calculated within each age group and presented as number (percentage).

© 2021 Cheng E et al. *JAMA Network Open.*

eTable 2. Hazard Ratios (95% CIs) of Age at Diagnosis With Overall Mortality[a]

Age	Unadjusted	Model 1[b]	Model 2[c]	Model 3[d]
<20	1.03 (0.88-1.20)	1.21 (1.03-1.42)	1.05 (0.89-1.23)	1.07 (0.91-1.25)
20 - 24	1.29 (1.17-1.41)	1.27 (1.16-1.39)	1.14 (1.04-1.25)	1.16 (1.06-1.28)
25 - 29	1.32 (1.24-1.41)	1.13 (1.06-1.21)	1.14 (1.06-1.21)	1.06 (1.00-1.14)
30 - 34	1.29 (1.23-1.35)	1.06 (1.00-1.11)	1.14 (1.08-1.20)	1.03 (0.98-1.08)
35 - 39	1.22 (1.18-1.28)	1.02 (0.98-1.06)	1.10 (1.05-1.15)	1.00 (0.96-1.04)
40	1.20 (1.13-1.28)	1.04 (0.98-1.10)	1.17 (1.10-1.24)	1.09 (1.03-1.16)
41	1.21 (1.14-1.28)	1.04 (0.98-1.10)	1.17 (1.10-1.24)	1.09 (1.03-1.15)
42	1.26 (1.20-1.33)	1.08 (1.02-1.14)	1.21 (1.14-1.28)	1.12 (1.06-1.19)
43	1.27 (1.20-1.34)	1.06 (1.01-1.12)	1.20 (1.14-1.27)	1.10 (1.04-1.16)
44	1.31 (1.25-1.38)	1.11 (1.06-1.17)	1.25 (1.19-1.31)	1.15 (1.09-1.21)
45	1.28 (1.22-1.34)	1.09 (1.04-1.15)	1.22 (1.16-1.28)	1.13 (1.07-1.18)
46	1.28 (1.22-1.34)	1.09 (1.04-1.14)	1.21 (1.16-1.27)	1.12 (1.07-1.17)
47	1.28 (1.23-1.34)	1.10 (1.06-1.16)	1.22 (1.17-1.28)	1.13 (1.08-1.18)
48	1.31 (1.25-1.37)	1.13 (1.08-1.18)	1.22 (1.17-1.28)	1.13 (1.08-1.18)
49	1.31 (1.26-1.37)	1.15 (1.10-1.20)	1.23 (1.18-1.29)	1.16 (1.11-1.21)
50	1.00	1.00	1.00	1.00
51	1.14 (1.09-1.19)	1.12 (1.08-1.17)	1.11 (1.07-1.16)	1.11 (1.06-1.15)
52	1.18 (1.13-1.23)	1.14 (1.10-1.19)	1.12 (1.08-1.17)	1.11 (1.07-1.16)
53	1.24 (1.19-1.29)	1.19 (1.14-1.24)	1.16 (1.12-1.21)	1.15 (1.10-1.20)
54	1.29 (1.24-1.34)	1.22 (1.18-1.27)	1.21 (1.16-1.26)	1.18 (1.14-1.23)
55	1.30 (1.25-1.35)	1.25 (1.20-1.30)	1.21 (1.16-1.26)	1.20 (1.15-1.24)
56	1.35 (1.30-1.40)	1.29 (1.24-1.34)	1.25 (1.20-1.30)	1.22 (1.17-1.27)
57	1.38 (1.33-1.43)	1.30 (1.25-1.35)	1.27 (1.22-1.32)	1.23 (1.18-1.28)
58	1.41 (1.36-1.47)	1.36 (1.31-1.41)	1.29 (1.24-1.34)	1.28 (1.23-1.32)
59	1.44 (1.39-1.49)	1.42 (1.36-1.47)	1.34 (1.29-1.39)	1.33 (1.28-1.38)
60 - 64	1.52 (1.47-1.57)	1.52 (1.47-1.56)	1.38 (1.34-1.43)	1.38 (1.34-1.43)
65 - 69	1.60 (1.56-1.65)	1.73 (1.67-1.78)	1.37 (1.33-1.42)	1.41 (1.37-1.46)
70 - 74	1.91 (1.85-1.97)	2.18 (2.12-2.25)	1.63 (1.58-1.68)	1.70 (1.65-1.76)

© 2021 Cheng E et al. *JAMA Network Open.*

75 - 79	2.37 (2.30-2.44)	2.89 (2.80-2.98)	2.07 (2.00-2.14)	2.17 (2.10-2.24)
≥80	3.71 (3.60-3.82)	4.89 (4.75-5.04)	3.26 (3.16-3.37)	3.27 (3.17-3.38)

[a] Demographic characteristics including sex, race, geographic location, and residence setting. Socioeconomical status including median income in zip code of residence by quartiles, percentage of residents by zip code graduating from high school, and primary health insurance. Clinical factors including stage, tumor location, and Charlson-Deyo comorbidity score. Treatment factors including facility type, surgery, radiation, chemotherapy, and immunotherapy.

[b] Adjusted for stage.

[c] Adjusted for demographic characteristics, socioeconomical status, clinical factors (not including stage), and treatment factors.

[d] Adjusted for demographic characteristics, socioeconomical status, clinical factors (including stage), and treatment factors.

© 2021 Cheng E et al. *JAMA Network Open.*

eFigure 2. Associations of Age at Diagnosis With Overall Mortality

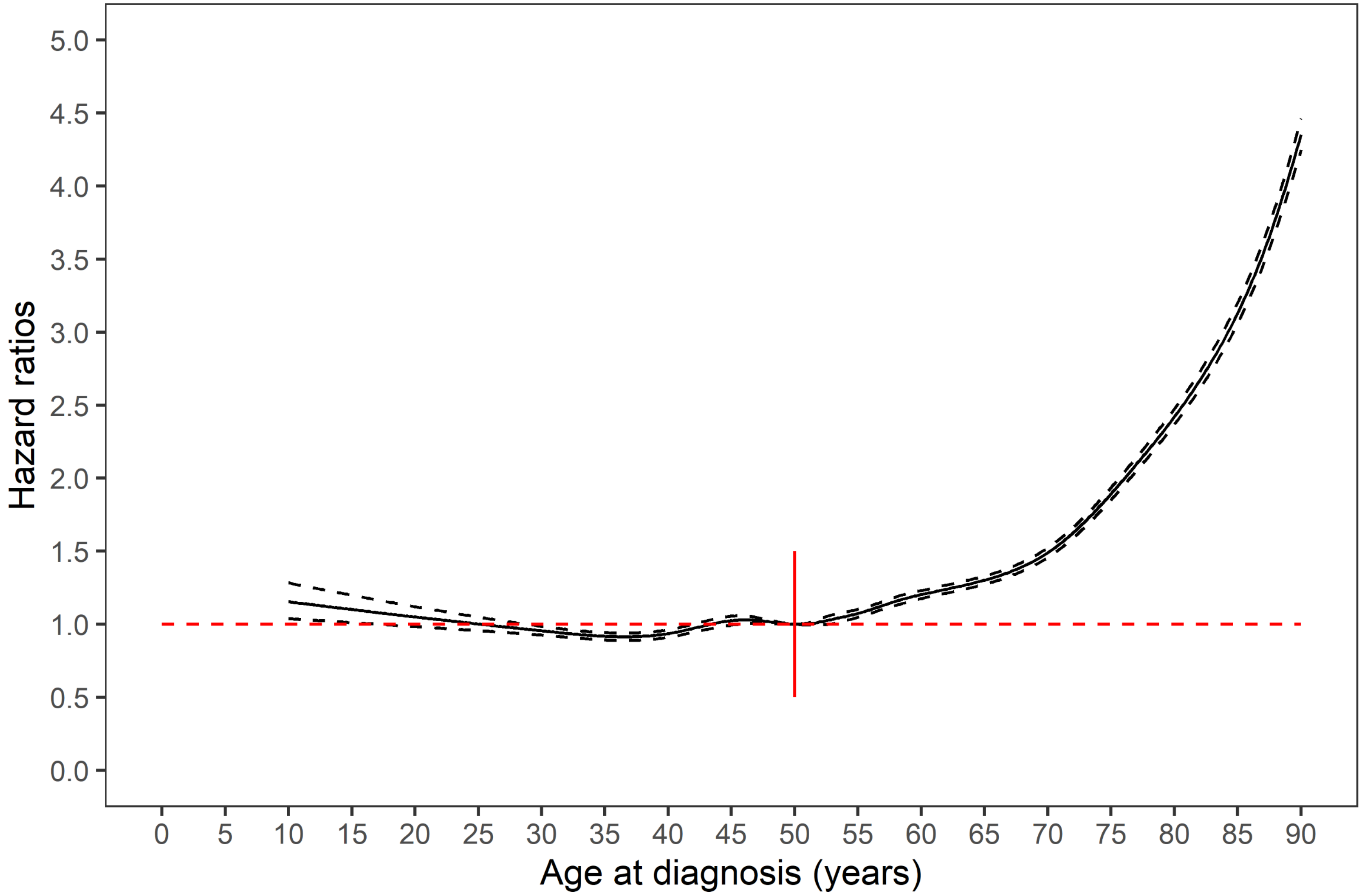

The hazard ratios of age at diagnosis were calculated using restricted cubic spline regression. Point estimates of hazards ratios for each age were plotted as the solid line and 95% confidence intervals were displayed as dotted lines.

© 2021 Cheng E et al. *JAMA Network Open.*

eTable 3. Survival Rates (% With 95% CIs) of Early-Onset and Later-Onset Colorectal Cancer by Stage

	**Early-onset CRC**					**Later-onset CRC**			
	1 Year	3 Years	5 Years	10 Years		1 Year	3 Years	5 Years	10 Years
Overall	90.8 (90.6, 91.0)	72.8 (72.5, 73.1)	63.2 (62.8, 63.5)	53.6 (53.2, 54.0)		90.1 (89.8, 90.3)	74.2 (73.8, 74.5)	64.9 (64.5, 65.3)	54.3 (53.8, 54.8)
Stage									
I	98.9 (98.7, 99.0)	96.5 (96.2, 96.8)	93.7 (93.2, 94.1)	86.7 (85.9, 87.5)		98.5 (98.3, 98.7)	95.7 (95.4, 96.0)	92.4 (92.0, 92.8)	83.8 (82.9, 84.6)
II	97.9 (97.6, 98.0)	91.2 (90.8, 91.6)	85.0 (84.4, 85.5)	75.6 (74.8, 76.5)		96.6 (96.4, 96.9)	89.4 (88.9, 89.9)	82.1 (81.4, 82.7)	70.1 (69.1, 71.2)
III	96.2 (96.0, 96.4)	82.4 (82.0, 82.9)	72.0 (71.4, 72.6)	60.0 (59.2, 60.8)		95.4 (95.1, 95.6)	81.8 (81.3, 82.4)	71.0 (70.3, 71.7)	57.3 (56.3, 58.2)
IV	73.9 (73.4, 74.4)	33.6 (33.1, 34.2)	18.7 (18.2, 19.3)	10.3 (9.8, 10.9)		69.3 (68.6, 69.9)	30.7 (30.0, 31.4)	16.2 (15.6, 16.8)	8.7 (8.1, 9.3)

© 2021 Cheng E et al. *JAMA Network Open.*

eTable 4. Multivariable[a] Hazard Ratios Comparing Early-Onset vs. Later-Onset CRC Colorectal Cancer by Stage

	HR (95% CI)	*P*-value for interaction[b]
Stage		<0.001
I	0.87 (0.81-0.93)	
II	0.86 (0.82-0.90)	
III	0.98 (0.95-1.01)	
IV	0.96 (0.94-0.98)	

Abbreviations: CRC, colorectal cancer; HR, hazard ratio; CI, confidence interval

[a] Adjusted for demographic characteristics (sex, race, geographic location, and residence setting), socioeconomical status (median income in zip code of residence by quartiles, percentage of residents by zip code graduating from high school, and primary health insurance), clinical factors (stage, tumor location, and Charlson-Deyo comorbidity score), and treatment factors (facility type, surgery, radiation, chemotherapy, and immunotherapy).

[b] P-values were assessed using maximum likelihood tests.

© 2021 Cheng E et al. *JAMA Network Open.*
